# Intermittent Suckling Causes a Transient Increase in Cortisol That Does Not Appear to Compromise Selected Measures of Piglet Welfare and Stress ^†^

**DOI:** 10.3390/ani6030024

**Published:** 2016-03-17

**Authors:** Diana L. Turpin, Pieter Langendijk, Tai-Yuan Chen, David Lines, John R. Pluske

**Affiliations:** 1 School of Veterinary and Life Sciences, Murdoch University, Murdoch WA 6150, Australia; J.Pluske@murdoch.edu.au; 2 South Australian Research and Development Institute, Roseworthy Campus, JS Davis Building, Roseworthy SA 5371, Australia; Pieter.Langendijk@trouwnutrition.com (P.L.); Tai.Chen@sa.gov.au (T.-Y.C.); 3 Sunpork Farms, Wasleys SA 5400, Australia; dlines@austporkfarms.com.au

**Keywords:** pig, weaning, intermittent suckling, welfare, stress

## Abstract

**Simple Summary:**

This study assessed the effects intermittent suckling (IS) had on physiological and behavioral indices of piglets before and after weaning. Piglets were allocated to either a control treatment (conventional weaning) or an IS treatment (separation from the sow for 8 h per day starting the week before weaning). Apart from an initial peak in cortisol at the start of IS, piglets subjected to IS did not show physiological changes suggestive of a chronic stress response before and after weaning. The event of weaning still caused a decrease in growth rate and an increase in white blood cell parameters in both treatment groups. However, the IS piglets tended to gain more weight in the second half of the week after weaning. The results of this study suggest that short periods of separation (e.g., 8 h/day) do not appear to compromise piglet welfare over the peri-weaning period.

**Abstract:**

This study tested the hypothesis that piglets subjected to intermittent suckling (IS) would show changes in physiological and behavioral indices indicative of compromised welfare in the peri-weaning period. A total of 21 primiparous sows and their litters were allocated to either a control treatment (*n* = 10) where piglets were weaned conventionally, or an IS treatment (*n* = 11) where piglets were separated daily from their sows for 8 h starting the week before weaning. Performance, physiological and behavioral measures were taken at various time points during the week before and after weaning. Plasma cortisol levels were higher (*p* = 0.01) in IS piglets 7 d before weaning. Regardless of treatment, the N:L ratio at 3 d and 7 d after weaning was higher (*p <* 0.05) than that at 1 d before weaning. The IS piglets ate more creep feed during lactation (*p* < 0.05), and there was a tendency for the IS piglets to gain more weight between 3 d and 7 d after weaning (*p <* 0.1). This study showed that, aside from an increase in cortisol at the start of IS, piglets subjected to IS did not display physiological or behavioral changes indicative of compromised welfare.

## 1. Introduction

The induction of estrus and subsequent mating during lactation is an alternative reproduction system that is being explored by some pork industries around the world to reduce sow confinement. In this system, anestrus, which is usually experienced by the sow in lactation, is primarily overcome by the use of a limited nursing weaning regime such as intermittent suckling (IS) [1] in combination with boar exposure [2]. Intermittent suckling involves a temporary daily separation of the piglets from the sow during the latter part of lactation to reduce the suppressive effect that suckling has on luteinizing hormone secretion, which may allow ovulation to occur and subsequent conception during lactation [1]. Since one of the main stressors a piglet experiences under commercial conditions is weaning [3], IS regimes appear to have the benefit of mimicking the increasing amount of time sows would spend away from their piglets under more natural conditions, which has been shown to provide benefits to piglets after weaning [4,5]. Temporarily separating piglets from the sow before weaning allows piglets to become more familiar with solid food as an alternative nutrient source to milk, which causes increased solid feed intake before weaning and a subsequent improvement in growth after weaning [6,7,8,9]. However, the act of repeated maternal separation during lactation on aspects of the piglets’ stress response is of potential welfare concern since it is well established that maternal separation experienced at weaning is associated with altered behavior patterns such as aggression, belly nosing and higher vocalization rates [10], and a transient increase in adrenocorticotrophic hormone and cortisol most likely resulting from emotional stress of the mother-infant separation and exposure to a new environment [11]. Therefore, it is important to establish that short, repeated periods of separation during lactation do not make the event of weaning even more stressful.

The hypothesis tested in this study was that piglets separated from their dam for 8 h per day for 7 d in the week before weaning would show physiological and behavioral indices indicative of compromised welfare in the peri-weaning period. The study also examined the proposition that post-weaning performance would be superior in pigs subjected to IS before weaning compared with piglets from a conventional weaning regime due to increased creep feed intake.

## 2. Materials and Methods

The experimental design and procedures were approved by the Animal Ethics Committees of Primary Industries and Resources South Australia (25/13) and Murdoch University (N2650/14).

### 2.1. Animals, Experimental Design, Diets and Housing

The experiment was conducted on a commercial pig farm in South Australia. One block of 25 first-parity sows (Large White x Landrace) and their offspring were used. The number of piglets born alive varied from nine to thirteen with an average litter size of 10.3 ± 1.6 (mean ± SD). One sow was replaced with a foster sow due to poor udder development and three other sows were excluded from the trial due to low litter size, leaving a total of 21 sows and 216 piglets for use in the study.

At farrowing, sows and their litters were randomly allocated to one of two treatments: conventional weaning (CW) (control group) (*n* = 10 L) or intermittent suckling (IS) (*n =* 11 L). In total, 104 piglets were part of the CW treatment group and 112 piglets were part of the IS treatment group. The piglets in the CW treatment group remained with their sow continuously through the lactation period until weaning. The piglets in the IS treatment were separated from their sow and housed in a separation pen (see description below) for 8 h per day (07:00–15:00) for 7 d before weaning (average age at the start of separation was 22 ± 1.3 d; mean ± SD). Separation was achieved by ushering the piglets through an open gate between the crate and the separation pen. This process involved minimal handling and once the piglets had been moved, the gate was kept closed.

During lactation, the sows and piglets were housed in conventional farrowing pens (1. 7m × 2.4 m). The pens consisted of a covered, heated creep area for the piglets and a crate to individually house the sows (0.6 m × 2.4 m) while still allowing piglet access. A sow feeder and nipple drinker were located at the front of the crate. At the rear of each pen was a larger pen (1.7 m × 2 m) separated by a gate. This area was used to house the piglets during separation and was called the separation pen. Both the farrowing pen and separation pen had plastic, slatted flooring. Within 24 h of farrowing, piglets received a 1 mL IM iron injection (Feron 200+B12, 200 mg/mL iron dextran and 40 ug/mL cyanocobalamin; Bayer Healthcare, Pymble, Australia), a 2 mL IM injection of RespiSure One^®^ (*Mycoplasma hyopneumoniae* vaccine; Pfizer, West Ryde, Australia), and their tails were cut and cauterized. Sows were fed a standard lactation diet (9.14 MJ NE/kg; CP, 170 g/kg; standardised ileal digestible (SID) lysine, 8.7 g/kg) at 2.5 kg per day until and including the day of farrowing and then were fed *ad libitum* from the day after farrowing until weaning. All piglets were offered creep feed (9.6 MJ NE/kg; CP, 220 g/kg; crude fibre (CF), 34 g/kg; SID lysine, 13.3 g/kg) *ad libitum* from a rotary feeder with hopper (one feeder per farrowing crate) from 14 d before weaning. When piglets were separated from the sow, the rotary feeder with the creep feed was moved into the separation pen. An additional rotary feeder was used to supply water in the separation pen.

The average weaning age for both the control (CW) and IS treatment groups was 29.1 ± 1.8 d (mean ± SD). The piglets were transported a short distance from the farrowing building to the weaner building and sorted according to treatment, sex and size. There were eight weaner pens (1.7 m × 3.4 m) in total with an average of 24.8 ± 2.1 pigs per pen. The pens were divided into IS and CW treatment groups with small and large males and small and large females. Half of the pen floor was concrete with sawdust and the other half was slatted plastic. There was a nipple drinker at the back of the pen and a 5-hole weaner hopper on the side allowing pigs to have *ad libitum* access to weaner feed (9.6 MJ NE/kg; CP, 197 g/kg; CF, 40 g/kg; SID lysine, 11.4 g/kg). Gas heaters fitted to the roof were used to heat the room. At this time, pigs received an oral dose of Enterisol^®^Ileitis (Boehringer Ingelheim, St Joseph, MO, USA) and 1 mL IM CircoFLEX^®^ (porcine Circovirus associated disease vaccine; Boehringer Ingleheim Vetmedica, Berkshire, UK, RG128YS).

### 2.2. Measurements

Fourteen days before weaning, eight piglets per litter were made individually identifiable with numbered ear tags. Individual identification was used to trace litter groups and to monitor which piglets had been used for blood sampling (see details below). All piglets were weighed individually at 14 d, 7 d and 4 d before weaning and on the day of weaning. At 3 d and 7 d after weaning, a subsample of approximately 1–12 ear tagged pigs per weaner pen were randomly selected and individually weighed. Within the randomly selected subsample of pigs that were weighed after weaning, it was also ensured that four pigs per original litter group were represented. Creep feed disappearance from each feeder was measured on 7 d and 4 d before weaning, on the day of weaning and 3 d and 7 d after weaning. Creep feed that had disappeared from the feeder was considered eaten (there was little to no wastage).

An injury score, a subjective estimate of aggression, was collected from six randomly selected pigs per pen the day after weaning. The injury scoring system was adapted from Widowski *et al.* [12] and consisted of a four point scale for scratches and redness around the heads, ears, abdomen and flank. The injury score was assessed while the selected pigs were being weighed.

### 2.3. Blood and Plasma Analyses

At 7 d, 4 d and 1 d before weaning and 1 d, 3 d and 7 d after weaning, two piglets were randomly selected per litter and a blood sample was collected (piglets were not bled more than 2 times within a 7 day period). Blood sampling started at noon and it took approximately 2 h to sample 42 piglets. Therefore, for the days before weaning, sampling took place approximately 4 h to 6 h after separation of the IS piglets from the sow. Piglets were held in dorsal recumbency and blood samples were collected via jugular venipuncture with the procedure lasting no more than 90 seconds. The samples were collected in a lithium heparin coated tube and an EDTA coated tube. The EDTA samples were kept refrigerated overnight at 4 °C and the lithium heparin samples were centrifuged on the day of collection (20 min at 2800× g at 4 °C), and then 1ml of plasma aliquots were stored at −80 °C.

Plasma cortisol and corticotrophin releasing hormone (CRH) were determined using commercially available ELISA kits (Enzo Life Sciences, Cortisol ELISA kit, AD-901-071, Farmingdale, NY and BioSource Porcine Corticotrophin Releasing Hormone ELISA kit, MBS267253, San Diego, CA, USA) in accordance with the manufacturers’ instruction with the exception of the optical density for cortisol, which was read at 415 nm instead of the recommended 405 nm. The intra-assay CV for cortisol was 10.5% (low), 6.6% (medium) and 7.3% (high), and ≤8% for CRH.

White blood cell differential counts were measured electronically using a Cell Dyn 3700 (Abbotts, Macquarie Park, Australia). Plasma was analyzed at Animal Health Laboratories (Department of Agriculture and Food Western Australia) for the determination of haptoglobin.

### 2.4. Statistical Methods

Statistical analysis was performed using SPSS (v.21; IBM, St Leonards, Australia). All data were tested for normality using the Shapiro-Wilk value (>0.80 was considered normally distributed). The litter was the experimental unit for average daily feed intake (ADFI) before weaning as well as average daily gain (ADG) and body weight (BW) before and after weaning. The pen was the experimental unit for ADFI after weaning. To calculate the ADG from weaning to 3 d after weaning, the average BW of the litter at weaning was subtracted from the average BW of the four pigs representing the litter 3 d after weaning. This value was then divided by three to account for the 3 d difference between the measurements. Values for ADFI before weaning, cortisol and N:L ratios were negatively skewed, requiring transformation to force normality using log_10_ transformation before analysis. The mean values and confidence intervals were then back-transformed and expressed as least square means with 95% confidence intervals. Values for BW, ADG, ADFI and injury scores were analyzed using a general linear model with treatment as the fixed effect. Body weights at 14 d before weaning were also included as a covariate for the post-weaning BW and ADG analysis. A linear mixed model was used to analyze cortisol, N:L ratios, CRH and haptoglobin levels with sow identification and pen (after weaning only) fitted as random effects, and treatment and day as fixed effects. A day by treatment interaction was then used. Correlations involving ADFI were performed using the Pearson correlation test. Statistical significance was accepted at *p <* 0.05, and a trend was considered at *p* ≤ 0.1 and *p* ≥ 0.05.

## 3. Results

### 3.1. Piglet Mortality

Piglet pre-weaning mortality (from 14 d of the experiment to weaning) was 7.7% for CW piglets compared with 2.7% for IS piglets, however this difference was not significant (*p* > 0.1). There was no difference in post-weaning mortality (one week after weaning) between treatments (4.2% for CW *vs.* 4.6% for IS, *p =* 1.00).

### 3.2. Blood Measures Indicative of Stress and Welfare, and Injury Scores

**Figure 1:**
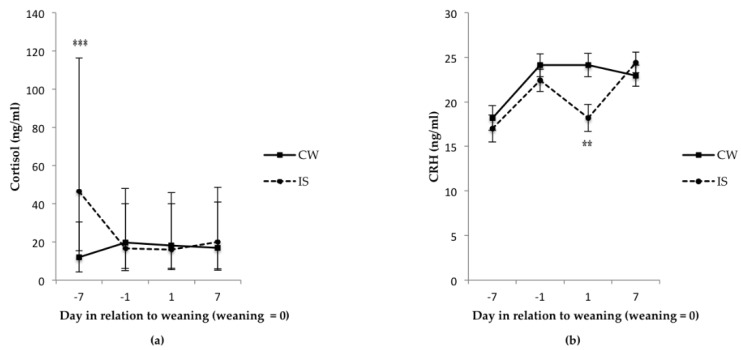
Effect of intermittent suckling (IS) on plasma (**a**) cortisol and (**b**) corticotropin releasing hormone (CRH) of piglets before and after weaning. ** Indicates differences between treatments (*p* < 0.01) per sampling day; *** indicates differences between treatments (*p <* 0.001); *n =* 20 for conventional weaning (CW) and *n* = 22 for IS. Plasma cortisol data were logarithmically transformed and then back transformed and expressed as least square means with 95% confidence intervals.

The IS treatment group had the highest cortisol concentration 7 d before weaning (the first day of separation) (Figure 1; *p* < 0.001), but this effect disappeared 6 d later. There was no treatment or day effect for cortisol concentration on 1 d and 7 d after weaning (Figure 1; *p* > 0.05). There was no difference in CRH concentration between the treatment groups at 7 d and 1 d before weaning (Figure 1; *p >* 0.05). However, one day after weaning, the IS group exhibited a reduction in CRH concentration resulting in a concentration that was lower than that of the CW pigs (Figure 1; *p* < 0.01). Seven days after weaning, the CRH concentration of the IS group increased again back to a similar concentration as the CW group (Figure 1; *p* > 0.05).

**Figure 2:**
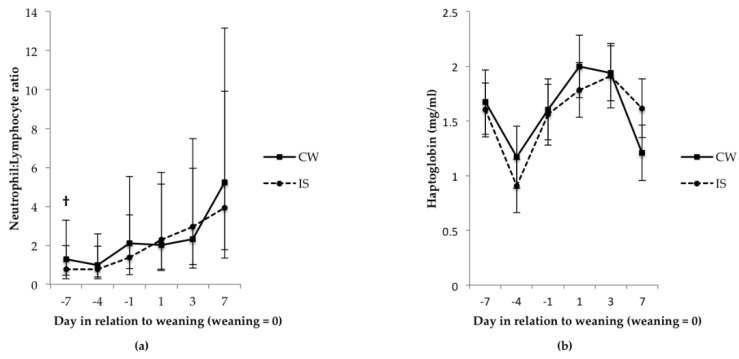
Effects of IS on plasma (**a**) N:L ratio and (**b**) haptoglobin of piglets before and after weaning. **†** Indicates trends between treatments (*p* < 0.1) per sampling day; *n =* 20 for CW and *n* = 22 for IS. Data for N:L ratios were were logarithmically transformed and then back transformed and expressed as least square means with 95% confidence intervals.

The CW litters tended to have a higher N:L ratio at 7 d before weaning compared with the IS litters (Figure 2; *p =* 0.1). There was no significant treatment effect for N:L ratio for the other time points up to 7 d after weaning. When treatments were combined, the N:L ratio on 3 d and 7 d after weaning was greater (*p <* 0.05) than the N:L ratio 1 d before weaning.

There was no difference in haptoglobin between treatments for any of the time points measured (Figure 2; *p* > 0.05). However, there was an overall day effect for haptoglobin with values at 4 d before weaning being lower (*p <* 0.05) than values on other measurement days except 7 d after weaning (*p >* 0.05). There was no significant treatment effect for incidence of scratches on the skin the day after weaning, with the scores being 2.0 ± 0.18 for CW *vs.* 2.3 ± 0.17 for IS (mean ± SD; *p* > 0.05). This was the same for redness measured on the same day; 0.70 ± 0.16 for CW *vs.* 0.96 ± 0.16 for IS (mean ± SD; *p* > 0.05).

### 3.3. Production Measures

There was no difference in piglet BW between the two treatment groups in the period from 14 d before weaning to 7 d after weaning (Table 1; *p >* 0.05). Piglet ADG did not differ (*p* > 0.05) between CW and IS litters before the start of IS, and there was also no treatment effect for ADG during the week of IS (7 d before weaning to weaning) (Table 2; *p* > 0.05). In the period from weaning to 3 d after weaning, both treatment groups exhibited a dramatic decrease in ADG, but there was no treatment effect (Table 2; *p >* 0.05). From 3 d to 7 d after weaning, IS pigs tended to have a higher ADG than the CW pigs (Table 2; *p* < 0.1).

Before the start of IS, there was no difference in the ADFI of creep feed between the two treatment groups (Table 2; *p* > 0.05). However, after the start of IS, creep ADFI was higher in IS litters in the period between 7 d and 4 d before weaning (*p* < 0.05) and tended to be higher in IS litters in the period from 4 d before weaning to weaning (*p <* 0.1) (Table 2). Average total creep feed intake during lactation was 291 ± 138.7 g/piglet (mean ± SD) for CW and 679 ± 125.4 g/piglet for IS (*p* = 0.05). However, creep ADFI was variable between the litters of different sows during lactation with total disappearance during lactation ranging from 98 g/piglet to 851 g/piglet in the CW litters, and 185 g/piglet to 1848 g/piglet in the IS litters. There was no treatment effect for ADFI when IS and CW pens were compared in the 7 d after weaning (147 ± 9.4 g/piglet/day for CW *vs.* 143 ± 9.4 g/piglet/day for IS; *p >* 0.05).

During lactation, there was a strong correlation between creep ADFI for the period 7 to 4 days before weaning and ADFI for the period from 4 d before weaning to weaning (*r* = 0.87; *p* < 0.001). However, no significant correlations existed between ADFI and ADG during the same time period. On the other hand, there was a positive correlation between ADFI during the entire lactation period and ADG between 3 d and 7 d after weaning (*r =* 0.47; *p* < 0.05).

## 4. Discussion

Intermittent suckling is a form of gradual weaning that can induce an estrus during lactation [13,14], allowing transfer of the mating management to the farrowing room and potentially reducing sow confinement after weaning. Previously, IS weaning regimes have shown improvements in the adaptation of piglets to weaning, and therefore arguably an improvement to welfare, through an increase in creep feed intake before weaning leading to an improvement in post-weaning performance [6,7,9]. However, there is concern that repeated maternal separation at a young age in lactation can cause emotional stress, compromising piglet welfare.

Plasma cortisol and CRH were used as physiological indicators of stress in the current study. However, and as defined by Moberg [15], stress is a biological response mechanism elicited when an individual perceives a threat to its homeostasis. When investigating the possible effects that IS has on piglet welfare, it is important to be able to identify when an animal is unable to physiologically or behaviorally cope with the challenge [15], *i.e.*, when there is a biological cost to the animal [16]. In the current study, there was an elevation in plasma cortisol after the first 4 h to 6 h of separation for the IS piglets compared to the CW piglets. This is consistent with previous studies that have looked at maternal separation in piglets [17,18] and other species [19], however it seems the IS piglets adapted to separation as cortisol values returned to the level of CW piglets by 6 d. To the authors’ knowledge, no other studies looking at IS have measured physiological stress responses during the separation period, however a behavior study by Berkeveld *et al*. [20] showed an increase in total litter activity in the IS piglets on the first day of separation, but this effect disappeared by the next measurement 2 d later. Despite the increase in activity reported by Berkeveld *et al.* [20], manipulative and aggressive behaviors associated with piglet distress in the same study were absent in IS litters during the separation period, but control litters experienced an increase in belly-nosing immediately after weaning. Although detailed behavior measurements were not taken in the current study, it seems that an increase in cortisol occurred at the same time (on the first day of separation) as the increased level of activity in the study by Berkeveld *et al.* [20], and in that study, the increase in activity was not associated with behaviors indicative of distress. In the current study, no differences between treatments were observed during the IS period with respect to growth, plasma CRH, blood N:L ratios and plasma haptoglobin levels. Therefore, it appears that IS piglets were able to adapt to the previously inexperienced separation from the sow with no overall biological cost to the animal suggesting that welfare, at least under the measures used in this study, was not compromised.

There was no day or treatment effect for cortisol after weaning when comparing values for the day before weaning to values for the period of 1 d and 7 d after weaning. The lack of a day effect is contrary to some weaning studies where an elevation in plasma, urinary or salivary cortisol was seen the day of or the day after weaning [21,22,23,24]. This lack of treatment effect in cortisol after weaning could be explained by the lack of difference in aggression between the groups after weaning as evidenced by the injury score results. Worsaae and Schmidt [25] showed that plasma cortisol concentrations in pigs can be positively correlated to post-weaning aggression, but since pigs in the current study were weaned at 29 days, they were perhaps less likely to develop behavior patterns associated with reduced welfare [26].

Similarly, there was also no day or treatment effect for haptoglobin over the weaning transition from 1 d before weaning to 7 d after weaning with all values sitting below the acute range of 3000 ug/mL to 8000 ug/mL [27]. The reason for the decrease in haptoglobin values at 4 d before weaning is not known. Haptoglobin is a major acute phase protein in the pig whose concentration in serum can vary in response to injury, infection, inflammation or stress [28,29]. Acute stress of less than 4 h is ineffective at altering haptoglobin levels [30]; however, an increase in haptoglobin can be seen 1 d to 3 d after an infectious insult [31,32,33]. Studies that measure the haptoglobin in response to weaning-related stressors which do not necessarily lead to health problems have shown an increase in haptoglobin at 7 d [34] and 10 d [35] after weaning compared to the day of weaning. This response was not seen in the current study, which suggests a lack of inflammatory response over the immediate post-weaning period, or it could also reflect the previously reported high level of variation in haptoglobin levels in apparently healthy pigs [27,36,37].

Despite no increase in cortisol or haptoglobin concentrations after weaning for both treatments, the increase in N:L ratios at 3 d and 7 d after weaning (when treatments were combined) in parallel to decreased ADG suggests that weaning was a stressful event. The immunological reactions that the stress of weaning can induce in pigs has been well documented [38,39,40,41], with studies also showing an increase in N:L ratio in the immediate post-weaning period [42,43]. However, the absence of a significant treatment effect in N:L ratios for all the time points after weaning suggests that exposure to IS before weaning does not have any further negative impact on the post-weaning immune response, as assessed by the N:L ratio.

Psychological stress has been linked to gastrointestinal tract dysfunction (GIT) [44,45]. In a study by Moeser *et al.* [24], the intestinal permeability of 19-day-old weaned piglets was increased compared with age-matched unweaned piglets, with the expression of corticotropin releasing hormone (CRH) receptors found to be increased in the intestinal tissue of the weaned pigs. In the same study, serum CRH levels seemed to reflect GIT disturbances more so than cortisol, suggesting that CRH may be a more sensitive indicator for stress induced intestinal dysfunction. The CRH results in the present study are not consistent with results from Moeser *et al.* [24] where weaning induced significant increases in serum CRH compared with unweaned controls. This lack of consistency may be an issue of weaning age since it seems this elevation in CRH over weaning is more pronounced at younger weaning ages (15 d to 19 d) than older ages (23 d to 28 d) [41,46]. It is interesting, however, that CRH concentrations in the current study are generally much higher than one would expect from weaning associated stress [24,41,46] and they do not follow the same pattern of expression as cortisol. The former could be an effect of sedation during venipuncture (piglets in the current study were conscious during venipuncture whereas piglets in the mentioned studies were sedated) and the latter may be related to a difference in timing of release from sites of origin. Furthermore, the reason for the significant decrease in CRH at 1 d after weaning for the IS pigs is also not known and since this treatment effect was not reflected in the post-weaning injury scores or other blood parameters, then it may be suggested that further research is required to examine the potential adaptive advantage gradual weaning may have on GIT morphology and function around the time of weaning.

Similar to other IS studies, the IS piglets in the current study ate double the amount of creep feed compared to the CW group before weaning. Nabuurs *et al.* [47] reported that piglets offered creep feed during lactation in combination with IS had an improved villous height and net absorption after weaning, suggesting that IS piglets were better adapted to weaning as a result of higher creep ADFI during lactation. The results of the current study could illustrate a similar effect as evidenced by the IS group tending to have faster growth than the CW group from 3 d to 7 d after weaning despite having similar ADFI during this time. This is further supported by the positive correlation between ADFI during lactation and ADG from 3 d to 7 d post-weaning, suggesting that litters that ate more during lactation were more likely to have faster growth rates between 3 d and 7 d post-weaning.

## 5. Conclusions

Apart from a transient peak in cortisol at the start of IS, piglets subjected to IS did not display neuroendocrine changes suggestive of a chronic stress response during the separation period (8 h daily separation for 7 d before weaning) and more importantly, there appeared to be no overall biological cost to these animals during this time as measured by performance, immune and inflammatory parameters. The event of weaning still caused a decrease in growth rate and an increase in N:L ratios in both groups. However, there was a tendency for IS pigs to grow faster than the CW pigs during the immediate post-weaning period. There were no differences in injury scores between the two groups after weaning.

## Figures and Tables

**Table 1:** Mean piglet body weight (kg) 14 days before weaning to 7 d after weaning for two different weaning regimens.

Day ^1^	Weaning Regimen ^2^		SEM	*p-*Value
	CW	IS		
−14	4.8	4.5	0.26	0.42
−7	6.4	6.0	0.34	0.44
−4	7.0	6.5	0.38	0.33
0	8.2	7.4	0.45	0.22
3	8.2	7.3	0.23	0.23
7	8.7	8.1	0.26	0.93

**^1^** Day in relation to weaning with 0 representing weaning (e.g., −14 is 14 d before weaning); **^2^** CW, conventional weaning; (*n* = 9); IS, intermittent suckling starting at day 21 (*n* = 11).

**Table 2:** Average daily gain (ADG) (grams per piglet) before and after weaning and average daily feed intake (ADFI) (grams per piglet) before weaning in two different weaning regimens.

Item	Weaning Regimen ^1^		SEM	*p-*Value
	CW	IS		
ADG (g)				
Before weaning				
Day −14 **^2^** to day −7	223	222	14.8	0.63
Day −7 to day −4	210	184	31.4	0.38
Day −4 to weaning	281	212	38.9	0.20
After weaning				
Weaning to day 3 **^3^**	2	−26	43.1	0.63
Day 3 to day 7 **^3^**	134	209	28.7	0.07
ADFI (g) **^4^**				
Day −14 to day −7	6 (3.81–10.59)	8 (4.85–12.22)		0.57
Day −7 to day −4	14 (7.24–26.42)	42 (22.54–77.09)		0.02
Day −4 to weaning	25 (12.30–50.12)	57 (30.27–106.41)		0.08

**^1^** CW, conventional weaning; (*n* = 9), intermittent suckling starting at 21 d (*n* = 11); **^2^** Days are expressed in relation to weaning (e.g., −14 is 14 d before weaning); **^3^** Growth of a subsample of four focus pigs per litter; **^4^** Data were logarithmically transformed before being subjected to the GLM. Values were then back-transformed and expressed as least square means with 95% confidence intervals (in parentheses).

## Abbreviations

The following abbreviations are used in this manuscript:

CRH: corticotrophin releasing hormone
CW: corticotrophin releasing hormone
IS: Intermittent suckling
